# Supplementary material for: Strain and Strain Rate Tensor Mapping of Medial Gastrocnemius at Submaximal Isometric Contraction and Three Ankle Angles
Source: Tomography. 2023 Apr 11;9(2):840–56. doi: 10.3390/tomography9020068 (PMC10143483; doi:10.3390/tomography9020068)
Supplement: Supplementary file 1 [file tomography-09-00068-s001.zip › SuppFigure S1.pdf]

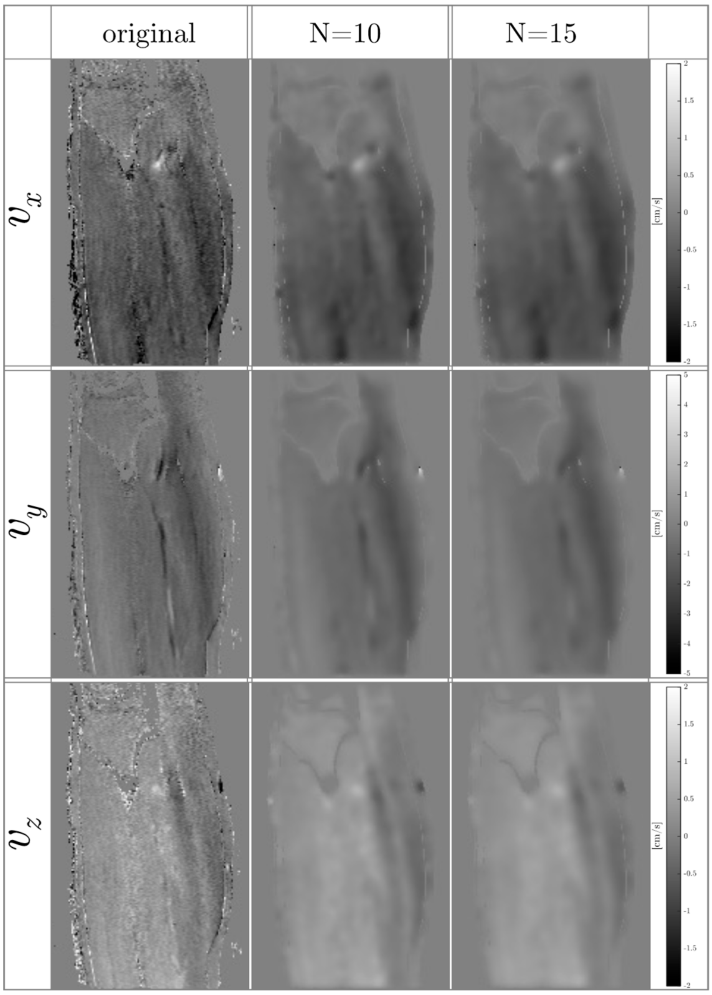

Figure S1: Saggital oblique phase images of the lower leg derived from velocity encoded along x-, y-, and z- directions are shown in the three rows respectively in the first column. The second and third columns show the corresponding images denoised with the anisotropic diffusion filter with iterations of N=10 (middle column) and N=15 (right column). The denoising is visually evident at both iterations while an increase in blurring is seen at the N=15 iteration.
