# Supplementary material for: Strain and Strain Rate Tensor Mapping of Medial Gastrocnemius at Submaximal Isometric Contraction and Three Ankle Angles
Source: Tomography. 2023 Apr 11;9(2):840–56. doi: 10.3390/tomography9020068 (PMC10143483; doi:10.3390/tomography9020068)
Supplement: Supplementary file 1 [file tomography-09-00068-s001.zip › SuppFigure S2.pdf]

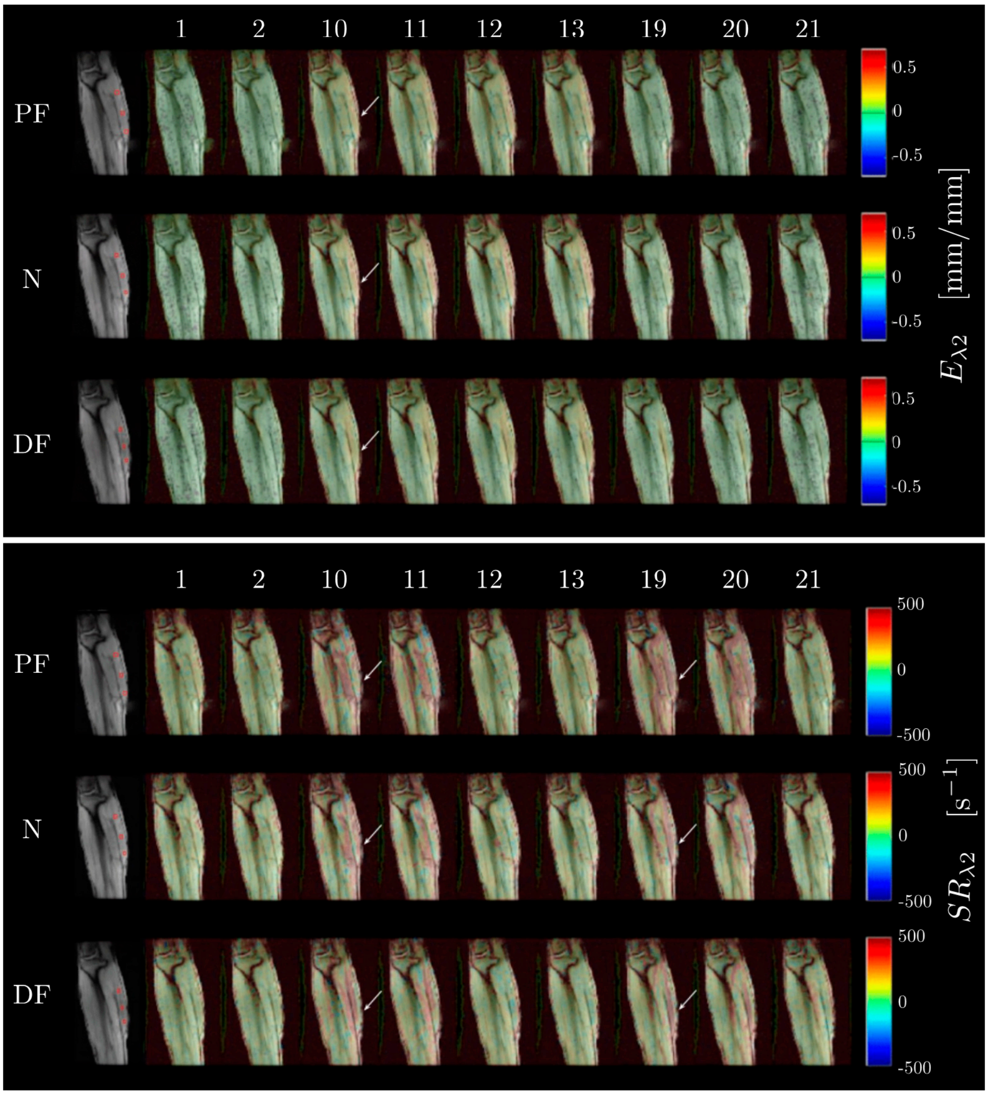

Figure S2: Maps of the positive strain ($E_{\lambda 2}$) (top panel) and positive strain rate ($SR_{\lambda 2}$) (bottom panel) projected on magnitude images at the corresponding temporal frame. Images shown here were acquired at 50% MVC, at each foot position PF, N, DF (order of rows top to bottom). Overlay allows for better identification of the underlying muscle, aponeuroses, and fascicles. The color maps are color-coded according to the legend with the figures. Select frames (from the acquired 22 dynamic frames) where peak strains and strain rates occur are shown here, the frame number is indicated on the top row. The peak of the strain occurs around frame 10-11 where the red shade corresponding to expansive strains in the MG and in the soleus can be seen (arrow points to the MG). The peak of the strain rate occurs in the contraction (~frame 7-8, arrow points to the MG) and relaxation (~frame 16) phases and this is visualized as red shades in the MG and in the soleus around these frames. The regions of interest in the MG (seen as red boxes in the first frame of each row) used to extract the deformation indices are shown here in the first frame.
