# Supplementary material for: Strain and Strain Rate Tensor Mapping of Medial Gastrocnemius at Submaximal Isometric Contraction and Three Ankle Angles
Source: Tomography. 2023 Apr 11;9(2):840–56. doi: 10.3390/tomography9020068 (PMC10143483; doi:10.3390/tomography9020068)
Supplement: Supplementary file 1 [file tomography-09-00068-s001.zip › SuppTable S1.pdf]

Table S1: Strain rate indices for different ankle angles and % MVC

Ankle Position	%MVC	Peak Force (N)	$SR_{\lambda 1}$ *	$SR_{\lambda 2}$	$SR_{out\text{-}plane}$	SR_{max} *
Plantarflexion	50	32 ± 6.959	-324.697 ± 29.449	183.309 ± 28.704	-26.378 ± 88.06	-254.003 ± 25.226
	25	16.72 ± 3.694	-241.874 ± 40.046	176.408 ± 63.533	-5.21 ± 43.613	-209.141 ± 50.796
Neutral	50	74.24 ± 9.655	-411.813 ± 37.634	246.392 ± 39.333	-11.343 ± 83.468	-329.102 ± 33.821
	25	37.68 ± 4.883	-324.148 ± 53.897	192.114 ± 47.256	-13.302 ± 75.636	-258.131 ± 41.771
Dorsiflexion	50	141.2 ± 9.246	-399.438 ± 68.12	261.62 ± 29.642	-40.484 ± 37.226	-330.529 ± 36.1
	25	73.82 ± 4.26	-252.608 ± 51.87	199.064 ± 26.901	-125.611 ± 74.794	-225.836 ± 35.655

* Significant difference between 25% and 50% MVC
