# Supplementary material for: Strain and Strain Rate Tensor Mapping of Medial Gastrocnemius at Submaximal Isometric Contraction and Three Ankle Angles
Source: Tomography. 2023 Apr 11;9(2):840–56. doi: 10.3390/tomography9020068 (PMC10143483; doi:10.3390/tomography9020068)
Supplement: Supplementary file 1 [file tomography-09-00068-s001.zip › SuppTable S2.pdf]

Table S2: Strain rate indices normalized to force for different ankle angles and % MVC

**Ankle Position**	**%MVC**	**Peak Force (N)**	$\mathbf{SR}_{\lambda 1}$ [*,δ]	$\mathbf{SR}_{\lambda 2}$ [*,δ,!]	$\mathbf{SR}_{out\text{-}plane}$	$\mathbf{SR}_{max}$ [o,!,*]
Plantarflexion	50	32 ± 6.96	-11.3904 ± 2.25	6.7902 ± 1.2145	-4.1554 ± 2.8026	-9.0903 ± 1.6516
	25	16.72 ± 3.69	-17.9205 ± 4.092	12.9672 ± 2.4176	-5.3091 ± 3.7725	-15.4439 ± 3.0423
Neutral	50	74.24 ± 9.65	-6.0078 ± 0.7691	3.6008 ± 0.52	0.9904 ± 1.3631	-4.8043 ± 0.5476
	25	37.68 ± 4.88	-10.2542 ± 2.1635	5.7975 ± 1.2654	0.6457 ± 3.4186	-8.0259 ± 1.26
Dorsiflexion	50	141.2 ± 9.25	-2.8763 ± 0.6285	1.9138 ± 0.1749	-0.3849 ± 0.2495	-2.3951 ± 0.3313
	25	73.82 ± 4.26	-3.8365 ± 0.7836	3.0488 ± 0.222	-2.4865 ± 0.8502	-3.4427 ± 0.4689

* Significant difference between 25% and 50% MVC

! Significant difference between PF and DF

δ Significant difference between PF and N

o Significant difference between N and DF
